# White matter structural topologic efficiency predicts individual resistance to sleep deprivation

**DOI:** 10.1111/cns.14349

**Published:** 2023-07-05

**Authors:** Fan Guo, Tian Zhang, Chen Wang, Ziliang Xu, Yingjuan Chang, Minwen Zheng, Peng Fang, Yuanqiang Zhu

**Affiliations:** ^1^ Department of Radiology, Xijing Hospital Air Force Medical University Xi'an China; ^2^ Department of Military Medical Psychology Air Force Medical University Xi'an China

**Keywords:** diffusion tensor imaging, sleep deprivation, vulnerability

## Abstract

**Background:**

Sleep deprivation (SD) is commonplace in modern society and there are large individual differences in the vulnerability to SD. We aim to identify the structural network differences based on diffusion tensor imaging (DTI) that contribute to the individual different vulnerability to SD.

**Methods:**

The number of psychomotor vigilance task (PVT) lapses was used to classify 49 healthy subjects on the basis of whether they were vulnerable or resistant to SD. DTI and graph theory approaches were used to investigate the topologic organization differences of the brain structural connectome between SD‐vulnerable and ‐resistant individuals. We measured the level of global efficiency and clustering in rich club and non‐rich club organizations.

**Results:**

We demonstrated that participants vulnerable to SD had less global efficiency, network strength, and local efficiency but longer shortest path length compared with participants resistant to SD. Lower efficiency was mainly distributed in the right insula, bilateral thalamus, bilateral frontal, temporal, and temporal lobes. Furthermore, a disrupted subnetwork was observed that consisted of widespread connections. Moreover, the vulnerable group showed significantly decreased strength of the rich club compared with the resistant group. The strength of rich club connectivity was found to be correlated negatively with PVT performance (*r* = −0.395, *p* = 0.005). We further tested the reliability of the results.

**Conclusion:**

The findings revealed that individual differences in resistance to SD are related to disrupted topologic efficiency connectome pattern, and our study may provide potential connectome‐based biomarkers for the early detection of the vulnerable degree to SD.

## Graphical abstract

Participants vulnerable to sleep deprivation had less global efficiency, network strength, and local efficiency but a longer shortest path length compared with the resistant group. Moreover, the vulnerable group showed significantly decreased strength in the rich club. The strength of rich club connectivity was negatively correlated with PVT.

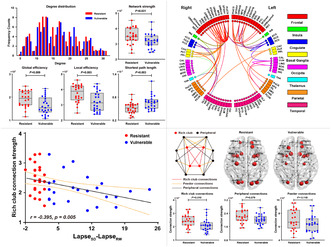

## 1 INTRODUCTION

Sleep is an important evolutionary conserved process for maintaining the body function and lack of sleep has severe health‐related consequences.^1^ Sleep plays an essential role in normal cognitive, affective, social, and physical functioning.^2^ Sleep deprivation (SD) is becoming increasingly common in our hectic society. Understanding the neurobiological mechanisms of SD is of great importance. Recent neuroimaging studies found that SD is accompanied by functional and structural disruption from both voxel and network perspective.^3^, ^4^, ^5^, ^6^ It has been found that individuals who suffered from SD may not experience the same extent of memory or attentional impairment. Although individual differences in vulnerability or resistance to SD are highly reproducible within a given individual, it has proven difficult to find a reliable predictive marker for the two groups.^7^ Many studies have focused on functional activation and structural pathways for describing the SD capacity. For instance, Chee et al.^8^ reported that SD‐vulnerable individuals showed reduced top‐down fronto‐parietal signal, while non‐vulnerable people showed a trend toward higher top‐down biasing of attention and preserved visual cortex activation during SD lapses. Our previous study also showed that white matter fibers connecting within the frontal–parietal attention network, the thalamus to the prefrontal cortex, and the left and right hemispheres contributed the most to classification accuracy between SD‐vulnerable and SD‐resistant participants.^9^ However, the neuronal mechanisms underlying the individual differences remain largely unclear, especially from the network perspective.

Graph analysis allows the quantitative analysis of network organization, describing the connections of the brain as a collection of nodes (brain regions) that communicate by connecting edges (white matter tracts) defined by diffusion tensor imaging (DTI).^10^ Network measures could assess how information is globally integrated.^11^ A “rich club” is formed by brain hubs of high‐degree nodes. The rich club is characterized by a tendency for hubs to be more densely connected among themselves than with peripheral regions.^12^ This organization provides important information on the higher‐level topology of the brain network.^13^ Recent study has demonstrated that the rich‐club architectures of default mode network and sensorimotor network were reconstructed after SD.^14^ But the resting state network disruption mechanisms of SD, and whether it could act as a classification biomarker of SD‐vulnerable and SD‐resistant groups remain unclear.

In the study, DTI data were collected to construct individual white matter structural networks. Graph analysis was applied to investigate the topologic alterations in the structural connectome in the SD individuals. The aim was to determine the different patterns of white matter connectivity and topologic alterations of the brain structural connectome in SD‐vulnerable or ‐resistant individuals.

## 2 MATERIALS AND METHODS

### 2.1 Subjects

Forty‐nine participants were recruited from universities and local community via advertisements. Consistent with our previous studies,^15^, ^16^ the exclusion criteria include sleep disorders, claustrophobia, drug/alcohol abuse, history of neurological or psychiatric illness. All participants were given informed consent approved by the clinical trial ethics committee of Xijing Hospital of the Air Force Medical University.

### 2.2 Sleep‐deprivation procedure

Participants made three visits to the laboratory. During the first visit, they were briefed about the experimental study protocol and provided a wrist Actiwatch (Philips Respironics, Mini Mitter) for further recording their sleep patterns. All participants signed the informed consent form. The second and the third visits were to undergo a magnetic resonance imaging (MRI) scan after normal sleep (Resting Wakefulness, RW) or after 24 h SD, and the last two visits were presented in a pseudo‐random order to minimize the influence of the scanning sequence. The time difference between the first visit and the second/third MRI visit was 1 week and was held constant for all participants. To avoid the persistent effect of SD, the interval between the last two visits was at least 1 week. The SD process began at 8:00 AM and ended at 8:00 AM on the following day. During SD, participants were allowed to read, watch TV or surf the internet while strenuous activities and beverages with caffeine were not allowed during the entire experiment. The temperature was around 23°C with standard light (340 lux). Two researchers accompanied to prevent the subjects from falling asleep.

Sustained attention performance was measured using the well‐defined psychomotor vigilance task (PVT), and detailed information was described in our previous research.^15^, ^16^ Briefly, participants were asked to press the space bar to stop the scrolling counter as quickly as possible. Reaction times longer than 500 ms were recorded as a lapse in performance. The participants completed the PVT immediately before MRI scanning during RW and SD conditions.

### 2.3 Data acquisition

Diffusion data were acquired using a GE Discovery MR750 3.0T scanner with a standard 8‐channel head coil at Xijing Hospital. During scanning, all participants were instructed to keep their eyes open, let the mind wander, and stay awake. Diffusion‐weighted sequences with single‐shot echo‐planar imaging aligned to the anterior–posterior commissural plane were acquired using the following parameters: field of view (FOV) = 282 mm × 282 mm, repetition time (TR)/echo time (TE) = 8000/89 ms, FA = 90°, slice thickness = 2.2 mm, and 62 continuous axial slices with no gap. The diffusion‐sensitizing gradients were applied along 64 non‐linear directions (*b* = 1000 s/mm^2^), combined with an acquisition without diffusion weighting (*b* = 0 s/mm^2^). The MRI scans were scheduled between 8:00 AM and 9:00 AM during the RW condition.

### 2.4 Data processing

All the steps for image processing were performed using ExploreDTI (www.exploredti.com).^17^ First, DTI images were realigned and corrected for eddy current distortions and head movements using the method described by Schilling et al.^18^ Second, whole‐brain tractography was performed in native space via fiber assignment by continuous tracking (FACT) algorithm.^19^ The tracking algorithm started at the center of the voxels with fractional anisotropy greater than 0.20 and ended when it reached a voxel with fractional anisotropy lower than 0.20 or when the angle between consecutive principal eigenvectors exceeded 45 degrees.

### 2.5 Network construction

In a white matter structural network, nodes and edges are the two fundamental elements. Nodes represent cortical and subcortical regions and edges correspond to white matter fiber bundles connecting distinct brain regions.

In this study, the whole brain was parcellated using the automatic anatomical label (AAL) template into 90 distinct and non‐overlapping regions (with the cerebellum excluded) to obtain network nodes.^20^ For each subject, the b0 image was linearly registered to the high‐resolution structural image using FLIRT (FMRIB's Linear Image Registration Tool).^21^ The structural image was then registered to the Montreal Neurological Institute (MNI) template using FMRIB's (Functional MRI of the Brain) Non‐linear Image Registration Tool (FNIRT).^22^ After combining those two transformations, the inverse transformation from the standard space to the native space can be obtained. The inverse transformation was applied to register the AAL template in the standard space to each subject's diffusion space.

Edges were defined as the white matter fibers connecting each pair of brain regions. In a weighted network, the weight of the edge was expressed as the number of connected fibers between the two regions. To control noise‐related false‐positive connections, weighted edges were threshed at 10. A weighted 90 × 90 White Matter (WM) structural network was constructed for each subject using this procedure.

### 2.6 Network measures

These graph metrics^10^ included: (1) network strength, computed as the average of the connection strength across all of the nodes in a network; (2) global efficiency, expressed as the average inverse shortest path length between two nodes; (3) local efficiency, computed as the average efficiency of local clusters consisting of the first neighbor of node; (4) shortest path length, defined as the average number of connections between nodes along the shortest paths in a network; (5) clustering coefficient, defined as the average likelihood that the neighbors of a node are interconnected.

In addition, the small‐world architecture of the network was also evaluated using the normalized clustering coefficient and normalized shortest path length.^23^ Normalized clustering coefficient was calculated by dividing the clustering coefficient of the networks with a set of random networks (*n* = 1000) with the same size and degree distribution as the real networks, as do normalized shortest path length.

### 2.7 Rich club measures

Rich club organization of complex networks described the phenomenon that the highly connected (high‐degree) hubs were more densely interconnected than was to be expected based on their high degree alone.^13^ Rich club regions as described throughout this study were selected on the basis of the group‐averaged backbone network. The backbone network was constructed by examining the significant nonzero connections across the subjects in each group by using a nonparametric one‐tailed sign test (*p* < 0.05, corrected). The node degree was calculated by the backbone network. The nodes with a nodal degree at least 1 standard deviation above the mean nodal degree were identified as the rich club region.^24^ On the basis of the categorization of the nodes of the network into rich club and peripheral regions,^25^ the edges of the network were classified into rich club connections, linking two rich club nodes; feeder connections, linking rich club nodes to peripheral nodes; and local connections, linking two peripheral nodes (Figure 4). The connection strength of each connection type was calculated as the sum of the corresponding edge weights. The weighted rich club coefficient was measured as the sum of the weights of the *n* edges connecting a subset of rich club nodes divided by the sum of the set of the strongest *n* connections in the total network. Normalized rich club coefficients were computed as the ratio between the real rich club coefficient and the averaged rich club coefficients obtained from 1000 matched random networks. Normalized rich club coefficient exceeding 1 was indicative of a network with a rich club organization.

### 2.8 Statistical analysis

Data analysis of demographic characteristics and clinical scores was performed using the SPSS version 24.0 software (IBM Corp), with the significance threshold set at *p* < 0.05. The Kolmogorov–Smirnov test was used to test for the normality of clinical and imaging metric data. Data that do not meet a normal/Gaussian distribution were analyzed using non‐parametric test. The categorical variables were examined using Chi‐square tests, and those for continuous variables were examined using two‐sample *t* tests for independent samples. The number of lapses from PVT task was analyzed by using the rank‐sum test because of the abnormal distribution. Between‐group differences in the graph parameters were examined for statistical significance using two‐sample *t* tests. For node properties of the network, multiple comparisons were corrected with the false discovery rate (FDR) correction. Since the two groups were age‐ and sex‐matched, additional correction for age and sex was not performed. Spearman rank correlation was calculated to evaluate the relation between affected network parameters and the number of lapses.

### 2.9 Test–retest analysis

To evaluate the reproducibility of the present findings, the WM networks constructed using different thresholds of fiber number (*W*_ij_ = 0, 10, 50, 100) and parcellation schemes (the Human Brainnetome Atlas) were further analyzed. In addition, taking into consideration the effect of region size on the reconstructed fiber number, the ROI corrected WM networks (fiber number divided by the mean volume of the connected regions) were also computed and analyzed. The reproducibility analysis details are described in Supplementary Material.

## 3 RESULTS

### 3.1 Demographic and behavioral measures

Descriptive statistics for demographic and sleep‐pattern information for the resistant and the vulnerable groups are shown in Table 1. There were no differences in age, gender, body mass index, and objective sleep measures between groups (all, *p* > 0.5). However, the SD‐resistant individuals had significantly lower scores in the average change in PVT lapse compared with the SD‐vulnerable individuals (*p* < 0.001; Table 1). Besides, we also calculated the head motions, since the tractography study is heavily influenced by head motions, as shown in Figure S1, no differences were found between the two groups.

**TABLE 1:** Demographic characteristics, Stanford sleepiness scale, and objective sleep measures.

	Vulnerable	Resilience	Statistical	*p* Value
	(*n* = 25)	(*n* = 24)	Value	
Gender (male/female)	12/13	12/12	0.02^a^	0.88
Age (years)	22.08 ± 1.77	22.00 ± 1.71	0.16^b^	0.87
Body mass index	23.70 ± 1.10	23.60 ± 0.96	0.33^b^	0.73
*Objective sleep characteristics from Actiwatch*				
Number of wakening each night	27.64 ± 6.28	27.95 ± 6.03	−0.18^b^	0.85
Sleep duration all night	6.75 ± 1.38	6.66 ± 1.28	0.23^b^	0.81
Night sleep durations before work days	6.35 ± 1.38	6.33 ± 1.27	0.05^b^	0.95
Night sleep durations before free days	7.14 ± 1.38	7.05 ± 1.28	0.25^b^	0.80
Sleep efficiency in %	84.20 ± 2.87	83.50 ± 2.02	0.98^b^	0.33
Sleep latency in minutes	16.32 ± 6.87	16.10 ± 7.63	0.11^b^	0.91
*PVT performance*				
Number of Lapse	9.56 ± 5.96	0.54 ± 1.06	−5.98^c^	<0.001

^a^ *T* value obtained by using the two‐sample *t* test.

^b^ Value from the χ^2^ test. *p* value also obtained by using the χ^2^ test.

^c^ *Z* value and *p* value obtained by using the rank‐sum test.

### 3.2 Graph theory analysis

**FIGURE 1:**
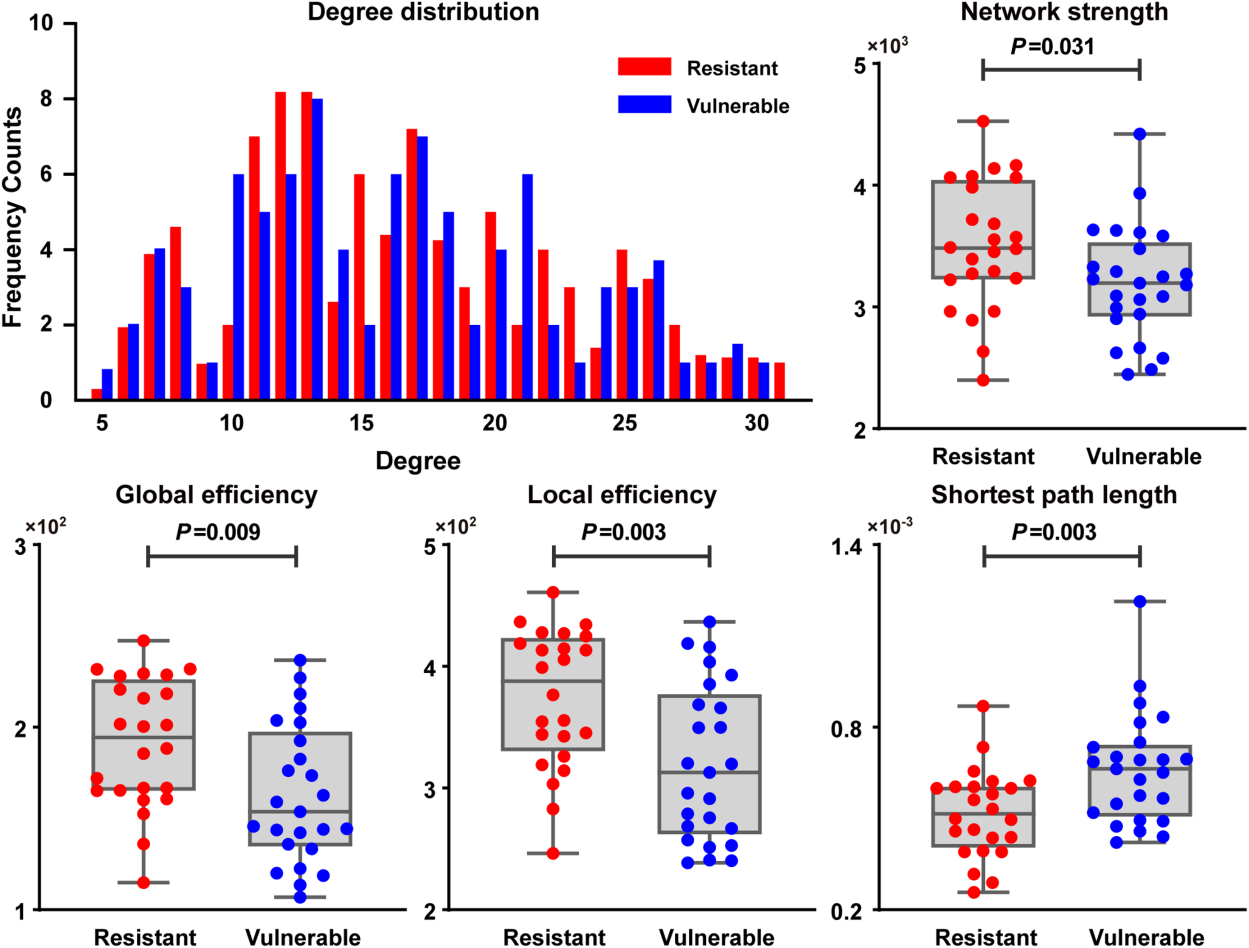
Degree distribution and group differences in the WM networks metrics of the vulnerable (*n* = 25) and resilient groups (*n* = 24).

The WM networks of both the resistant and the vulnerable groups showed a right‐skewed degree distribution, indicating that there were a small number of highly connected nodes (Figure 1). Both groups showed a characteristic small‐world topology (normalized clustering coefficient >1 and normalized shortest path length ≈ 1). Compared with the SD‐resistant individuals, the SD‐vulnerable individuals exhibited significantly less global efficiency (*t* = 2.73, *p* = 0.009), local efficiency (*t* = 3.16, *p* = 0.003), network strength (*t* = 2.22, *p* = 0.031), and a longer shortest path length (*t* = −3.10, *p* = 0.003; Figure 1). A trend was also observed for the clustering coefficient (*t* = 1.93, *p* = 0.06).

**FIGURE 2:**
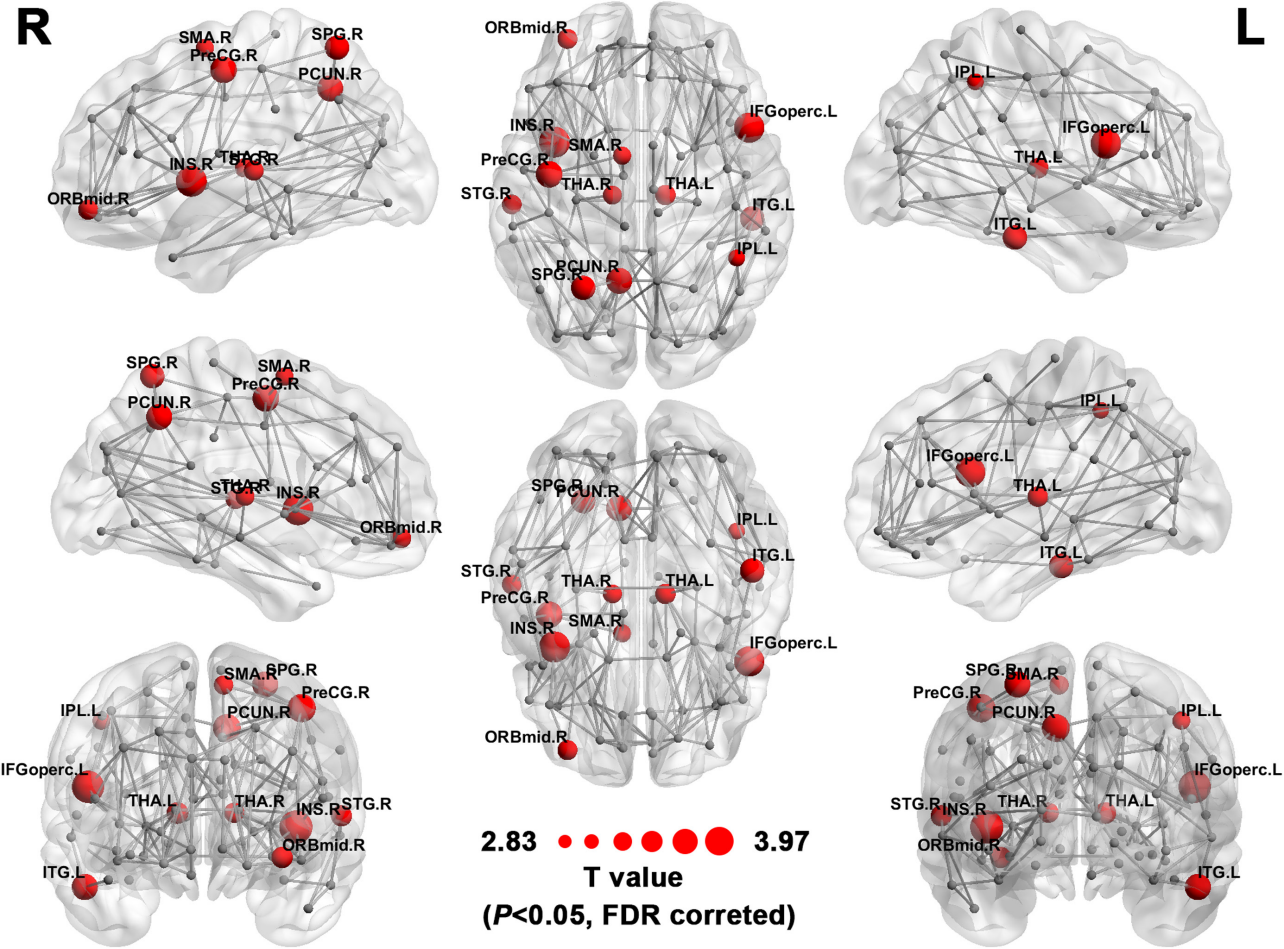
The distribution of the brain regions with significantly lower nodal efficiency in the vulnerable groups, left (L) and right (R). The regions with significant group differences in nodal efficiency are colored in red. The size of the node represents the significance of the between‐group differences. The network shown here was constructed by calculating the average of the WM network of all subjects and the threshold with a sparsity of 5%. IFGoperc, opercular part of inferior frontal gyrus; INS, insula; IPL, inferior parietal; ITG, inferior temporal gyrus; ORBmid, orbital part of middle frontal gyrus; PCUN, precuneus; PreCG, precentral gyrus; SMA, supplementary motor area; SPG, superior parietal gyrus; STG, superior temporal gyrus; THA, thalamus.

We identified the brain regions that showed significant between‐group differences in nodal efficiency (*p* < 0.05, FDR corrected). The vulnerable group showed decreased nodal efficiency, including the right insula, left inferior frontal gyrus, right precentral gyrus, right precuneus, left inferior temporal gyrus, right superior temporal gyrus, right superior parietal gyrus, bilateral thalamus, right middle frontal gyrus, right supplementary motor area, and left inferior parietal, compared with the resistant group (Figure 2 and Table 2).

**TABLE 2:** Nodal efficiency differences between resistant and vulnerable to SD group (*p* < 0.05 FDR correction).

Regions	Resilience (*n* = 24)	Vulnerable (*n* = 25)	*T* Value	*p* Value
INS.R	235.0 ± 46.9	184.4 ± 42.4	3.967	<0.001
IFGoperc.L	202.0 ± 42.4	150.3 ± 50.1	3.888	<0.001
PreCG.R	221.2 ± 53.2	170.3 ± 44.2	3.644	<0.001
PCUN.R	301.9 ± 70.0	233.4 ± 63.7	3.581	0.001
ITG.L	188.4 ± 46.0	145.8 ± 40.1	3.462	0.001
SPG.R	160.8 ± 40.2	121.2 ± 39.1	3.494	0.001
THA.L	222.9 ± 56.9	176.9 ± 46.0	3.121	0.003
ORBmid.R	209.6 ± 55.4	166.0 ± 42.6	3.099	0.003
STG.R	235.9 ± 60.3	180.9 ± 44.7	3.633	0.001
THA.R	225.9 ± 54.5	181.9 ± 47.2	3.023	0.004
SMA.R	203.4 ± 54.0	162.4 ± 43.3	2.936	0.005
IPL.L	159.9 ± 38.3	131.2 ± 32.5	2.833	0.007

*Note*: Data are presented as means ± standard deviation. The comparisons of nodal efficiency between groups were performed by using two‐sample *t* tests. *p* < 0.05 (FDR corrected) indicated a significant group difference.

Abbreviations: IFGoperc, opercular part of inferior frontal gyrus; INS, insula; IPL, inferior parietal; ITG, inferior temporal gyrus; ORBmid, orbital part of middle frontal gyrus; PCUN, precuneus; PreCG, precentral gyrus; SMA, supplementary motor area; SPG, superior parietal gyrus; STG, superior temporal gyrus; THA, thalamus.

### 3.3 Network‐based statistics

**FIGURE 3:**
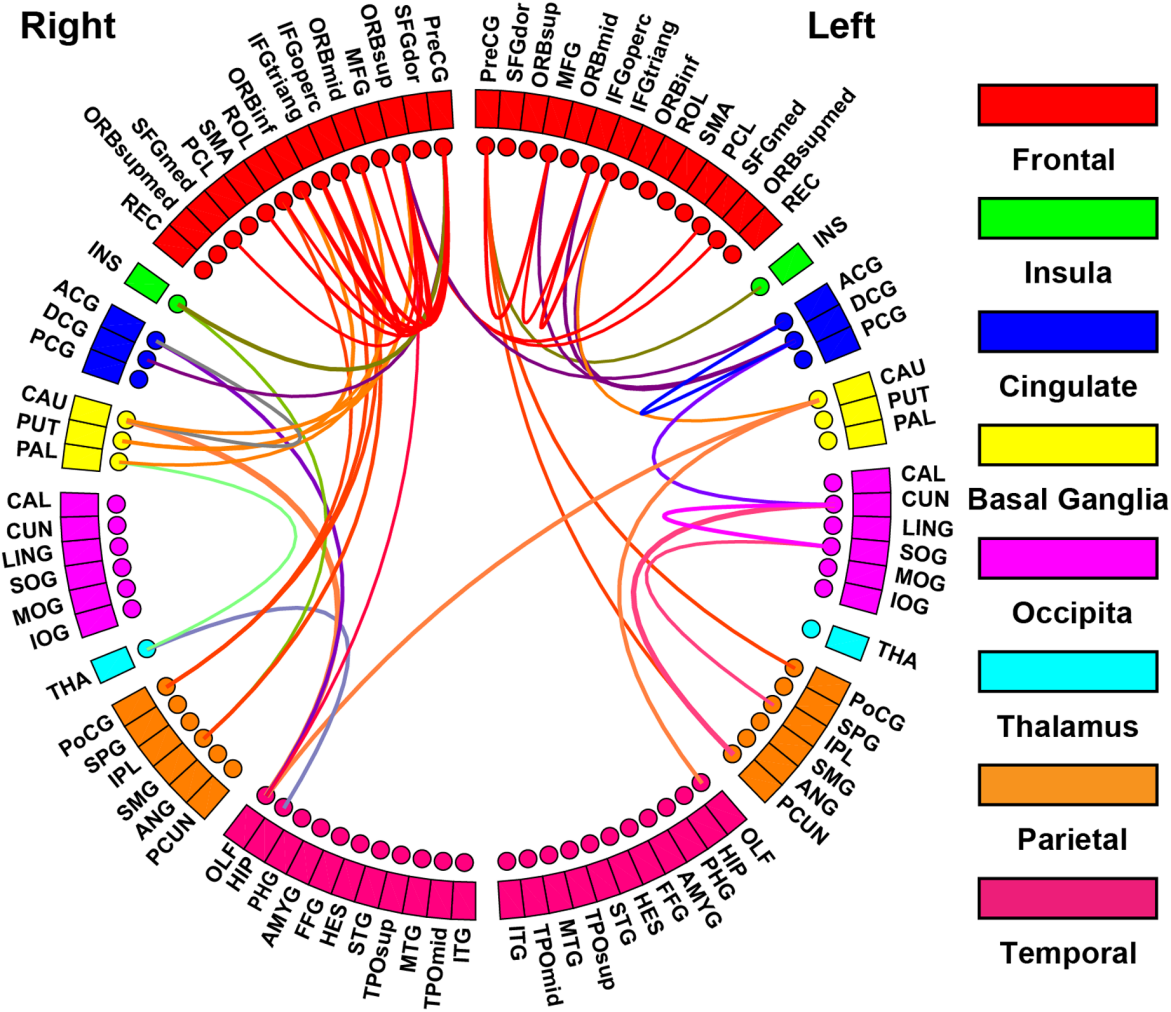
The aberrant connections in the vulnerable group relative to the resilient group.

We used the NBS method to identify the different structural connectivity between the vulnerable and resistant groups, and found that a single connected sub‐network (component) was significantly decreased (*p* < 0.05, non‐parametric permutation tests) in the vulnerable group. This network is widely distributed, involving 47 edges between 37 unique nodes, including both long‐ and short‐range connections. The connection of differences predominantly involved connections between the frontal cortex (14/47) and connections of the frontal cortex and parietal, basal ganglia, and insular regions (19/47; Figure 3). Remaining connections involved parietal and temporal pathways to basal ganglia, cingulate, occipital, and thalamus regions (Figure 3).

### 3.4 Rich club organization

**FIGURE 4:**
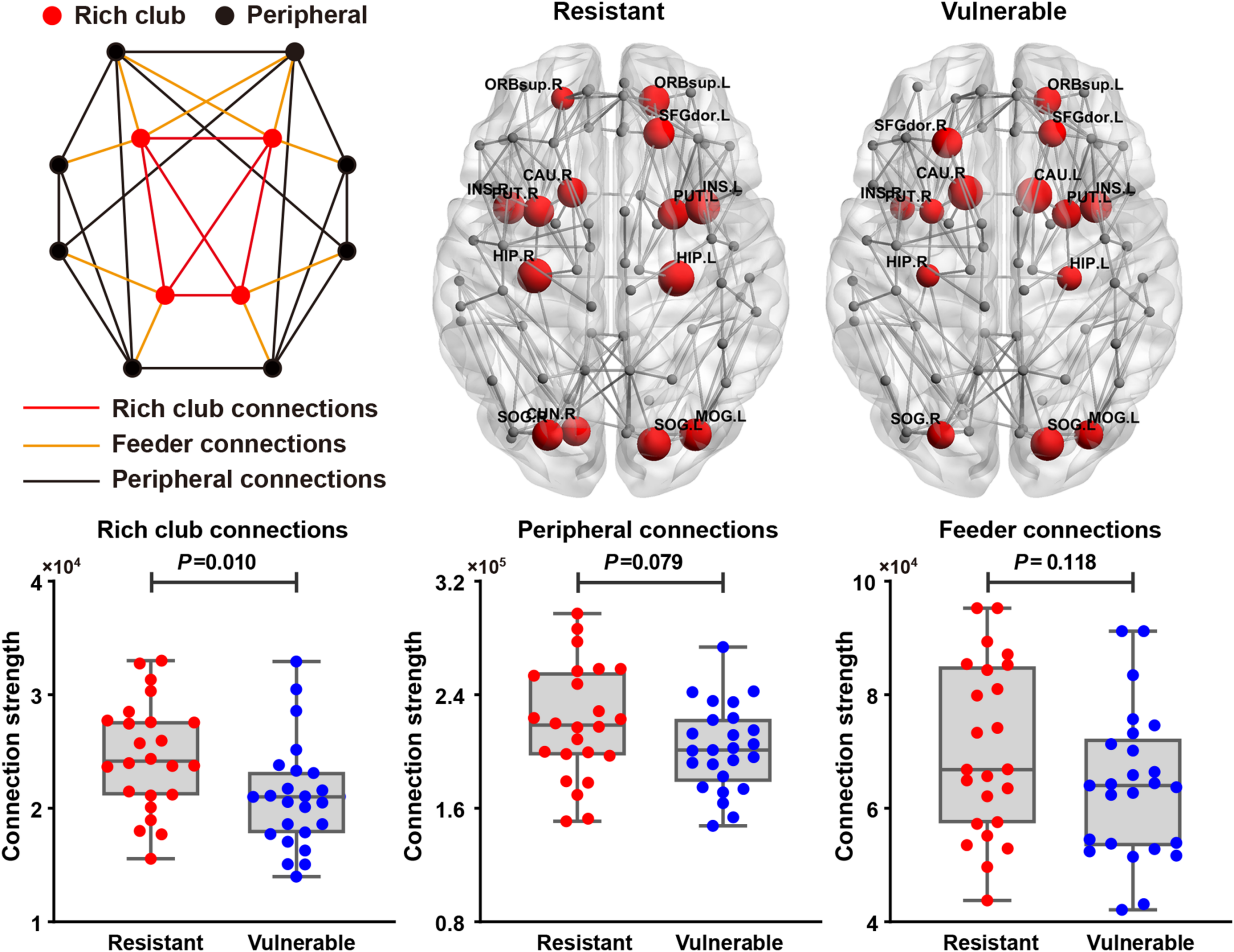
A simplified schematic of the rich club organization, including rich club connections, feeder connections, and peripheral connections. The hub distributions of the WM networks in the vulnerable and resilient groups. The hub nodes are marked in red, and the size of the node represents the strength of the connection. The network shown here was constructed by calculating the average of the WM network of all subjects and the threshold with a sparsity of 5%. Group differences in the rich‐club, feeder, and peripheral connection strengths. The bar and error bars represent the mean values and standard deviation of the network properties in each group, respectively. CAU, Caudate nucleus; HIP, Hippocampus; INS, insula; MOG, middle occipital gyrus; ORBsup, orbital part of superior frontal gyrus; PUT, putamen; SFGdor, dorsolateral part of superior frontal gyrus; SOG, superior occipital gyrus.

On the basis of the network of each group, similar hub distributions were found in both the vulnerable and resistant groups, mainly located in bilateral insula, lenticular nucleus, hippocampus, superior occipital gyrus, and left orbital superior frontal gyrus, dorsolateral superior frontal gyrus, middle occipital gyrus, and right caudate nucleus (Figure 4). However, hubs in the right orbital superior frontal gyrus and cuneus were only found in the resistant group. Furthermore, the left caudate nucleus was identified as hub only in the vulnerable group. Normalized‐rich club coefficients were greater than 1 for both the vulnerable (1.14 ± 0.06) and resistant (1.16 ± 0.05) groups. For the connection strength of the different categories of edges, the vulnerable group showed significantly decreased strength of the rich club compared with the resistant group (*t* = 2.67, *p* = 0.010), whereas there were no significant differences for the feeder and peripheral connection strength (Figure 4). Given the differential reduction in rich club connection strength, the ratio of rich club to feeder and of rich club to peripheral connection strengths between the two groups were also compared. Compared with the resistant group, the vulnerable group showed significantly lower ratios for rich club/feeder (*t* = 2.18, *p* = 0.034) and rich club/local connection (*t* = 2.72, *p* = 0.009).

**FIGURE 5:**
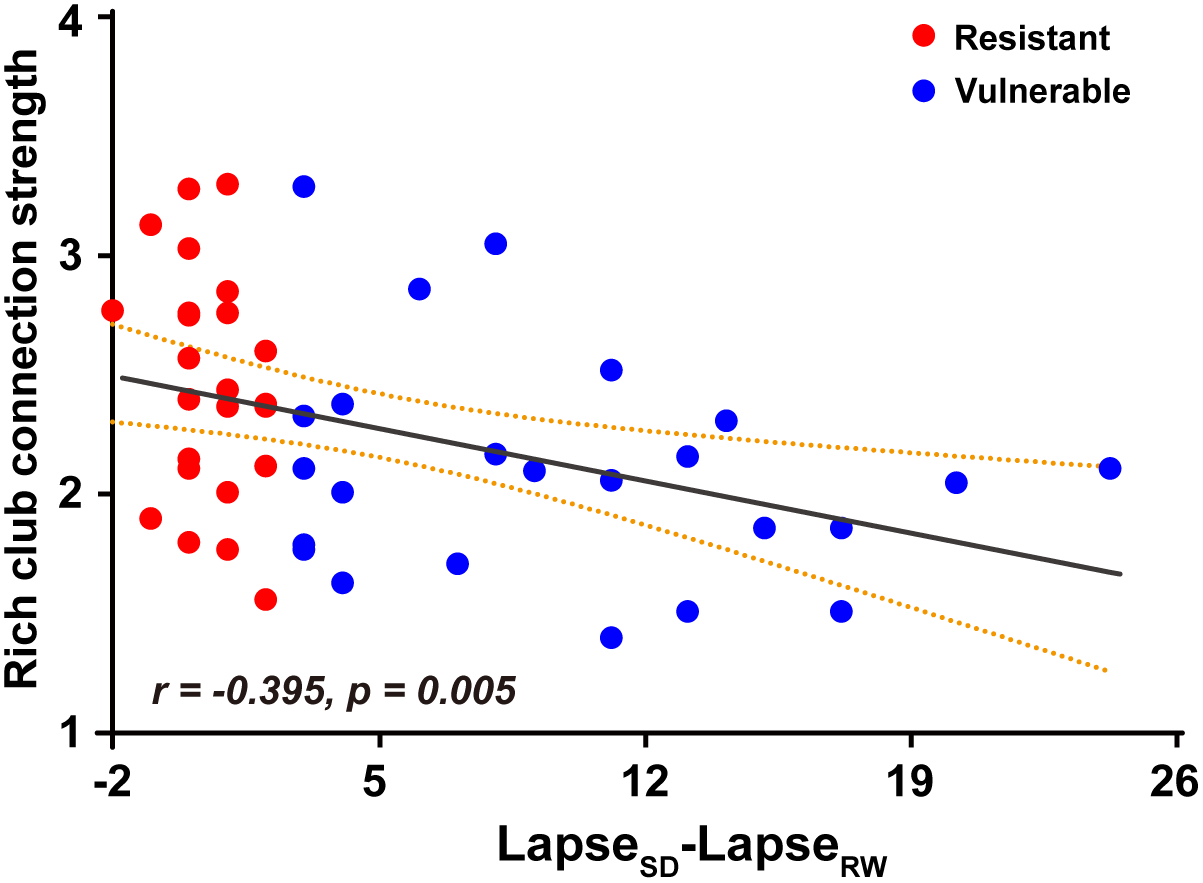
Relationship between the rich club connection strength and PVT performance.

The Spearman rank correlation revealed that the strength of the rich club was significantly negatively correlated with PVT performance (*r* = −0.395, *p* = 0.005) across all subjects, whereas the strength of the feeder and peripheral connections showed no significant correlation with PVT performance (Figure 5). Additionally, there were no significant correlations between the global and regional network metrics and PVT performance across all subjects.

### 3.5 Reproducibility of findings

To test the reliability of the results, we constructed individual WM networks with four different thresholds of fiber number (*W*_ij_ = 0, 10, 50, 100). For each threshold, similar group differences were found for network strength, global efficiency, local efficiency, and shortest path length (all *p* < 0.05; Figure S2). Except for the AAL template (90 ROIs), the Human Brainnetome Atlas (246 ROIs) was employed to evaluate the reproducibility of the current results. In our results, the vulnerable group exhibited significantly lower global efficiency (*t* = 3.01, *p* = 0.004), local efficiency (*t* = 3.90, *p* = 0.000), network strength (*t* = 2.79, *p* = 0.008), and a higher shortest path length (*t* = −2.43, *p* = 0.010) than the resistant group (Figure S3).

To exclude the potential effects of region size on the group differences of network metrics, we performed repeat statistical analyses on the network metrics calculated by the ROI‐corrected WM networks. Similar group differences in global efficiency (*t* = 3.15, *p* = 0.003), local efficiency (*t* = 3.00, *p* = 0.004), network strength (*t* = 2.46, *p* = 0.018), and shortest path length (*t* = −3.14, *p* = 0.003) were observed (Figure S4). Additionally, the vulnerable group showed decreased nodal efficiency, including the right supplementary motor area, left inferior parietal, bilateral thalamus, right inferior frontal gyrus, right precuneus, right insula, left inferior temporal gyrus, right superior parietal gyrus, right middle frontal gyrus, and right precentral gyrus (*p* < 0.05, FDR corrected; Figure S5 and Table S1).

## 4 DISCUSSION

In this study, we used DTI and topologic network analysis to explore the network differences in the white matter structural connections between SD‐vulnerable and ‐resistant individuals. Compared with resistant controls, vulnerable individuals to SD showed widespread disruption and reduced global and local efficiency, mainly located in the right insula, left opercular of inferior frontal gyrus, right precentral gyrus, right precuneus, left inferior temporal gyrus, right superior parietal gyrus, etc. Moreover, similar hub distributions and less connection strength were found in the SD vulnerable group compared with the resistant group. We demonstrated a reduced level of rich club connectivity in the vulnerable group. Importantly, the rich‐club strength changing was correlated with the PVT in the SD individuals, in that lower rich club connectivity strength was correlated with worse PVT performance. We showed the white matter network connectively was disrupted in SD‐vulnerable individuals, potentially adding effect on the SD vulnerability.

Sleep is of critical importance in maintaining health and well‐being. Lack of sleep could affect cognition in many ways and could negatively impact alertness, learning, memory, and executive function.^26^, ^27^ Growing evidence suggested that there were large individual differences in the vulnerability to SD. Searching for reliable markers for differentiating SD vulnerable and resistant individuals is of great importance. The human brain could be modeled as a network, known as connectome, and exhibits nontrivial topologic principles, such as small‐world properties, rich‐club organization, and modular structure.^28^ Kaufmann, et al. proved that SD strongly altered the connectivity of several resting‐state networks, including dorsal attention, default mode, and hippocampal networks.^1^ Pan et al.^14^ found the rich‐club architectures during cognitive tasks were reconstructed after SD, especially in the default mode network and sensorimotor network. Previous studies also indicated that white matter networks exhibited weaker rich‐club organization in primary insomnia patients than in healthy controls.^29^ However, few studies have focused on the topological connectivity alterations that contribute to the individual different vulnerability to SD.

From our study, the SD vulnerable group exhibited lower global and local efficiency of brain networks. The information transfer disruption may be caused by white matter degeneration at synaptic and neuronal levels previously proved by animal studies.^30^, ^31^ Neuroimaging studies also proved white matter, cortical thickness, and gray matter density changes.^32^, ^33^, ^34^ We further observed increased shortest path length in the vulnerable group compared with the resistant group. Specifically, the disrupted nodal efficiency was mainly located in the right insula, left opercular of inferior frontal gyrus, right precentral gyrus, right precuneus, left inferior temporal gyrus, and right superior parietal gyrus. Insula is one key area partially of the salience network and participated in attention and affective processes.^35^ Neuroimaging evidence suggested that the insula participates in the emotional experience after SD,^36^ and might be linked to inter‐individual differences in sleep and SD.^37^, ^38^ On the other hand, it is reported that the precuneus may be an important “distribution node” in the default mode network.^39^ And the previous study has indicated the default mode network may be a potential neuroimaging marker for differentiating vulnerable subjects from resistant subjects.^15^ Chee, et al. indicated that the superior parietal gyrus and inferior temporal gyrus were correlated with the performance accuracy decline from SD and thus might be useful for differentiating individuals who were vulnerable to SD.^40^, ^41^ Therefore, our result is consistent with the findings that highlight the potential role of insula, precuneus cortex, superior parietal gyrus, and inferior temporal gyrus in predicting vulnerability to SD.

The global disruption of structural connectivity was further observed by widely distributed edges with reduced connection strength in SD‐vulnerable participants. Widespread connection differences were found in several large white matter structures and short‐range connections including the brain cortex and the basal ganglia, cingulate, and thalamus regions. Our findings were consistent with emerging evidence of structural network abnormalities in SD‐vulnerable individuals, especially for the white matter connections within the frontal–parietal attention network, and the thalamus to prefrontal cortex.^9^, ^42^, ^43^

Similar hub distributions were found in both groups. However, hubs in the right orbital superior frontal gyrus and cuneus were only found in the resistant group. Furthermore, the left caudate nucleus was identified as a hub only in the vulnerable group. Hubs are important for global information transfer. The high degree and highly efficient hub nodes have higher blood flow, glucose metabolic rate, and in some diseases, important hubs were first affected by pathogenic factors.^24^, ^44^, ^45^ The rich club results also showed decreased connection strength, but not the feeder and peripheral connections. This white matter connectivity disruption and the hubs’ number decreasing might be the neural mechanisms that contribute to the vulnerability to SD, as might lead to cognitive impairment.^24^ The findings of reduced structural rich club connectivity consist of the evidence of structural network abnormalities in insomnia patients.^29^ However, our result was more tend for reflecting the different reactions of vulnerable or resistant individuals to short‐term effects of SD on the structure rather than the long‐term effect. A negative correlation between the rich club connection strength and PVT is also worth noting, indicating that the basic structural differences may lead to the disruption of the interactions between rich club regions, which confirmed our above‐mentioned speculation. Our study suggested that the structurally rich club connectivity might be sensitive for representing the individuals vulnerable to SD. However, we did not observe a correlation between either feeder or peripheral connections with PVT performance. Our findings hold significance in understanding and managing sleep deprivation effects. It provides valuable insights into risk mitigation strategies, particularly in professions demanding high alertness under sleep deprivation. Further, it can help guide the development of personalized education and training programs for individuals vulnerable to sleep deprivation, fostering improved sleep quality and attention maintenance strategies. The findings also have implications for mental health, facilitating early interventions in those showing cognitive impairment due to sleep deprivation. Our research could offer a basis for developing therapeutic interventions targeting specific brain structures, enhancing sustained attention under sleep deprivation. Several limitations of our study should be noted. First, the tractography is insufficient in resolving crossed fibers and may result in the loss of fibers. Second, we did not assess whether network changes occur as a consequence of SD or the cause of SD vulnerability. Longitudinal follow‐up studies are needed for clarifying the causal relationship between the PVT performances. Finally, the sample size was relatively small and the age distribution was relatively narrow.

In conclusion, our findings suggest that the vulnerable group is characterized by selectively impaired brain connectivity, and the degree or the disruption was related to the degree of SD. Network analysis may provide a useful disease marker to monitor the disease and study therapeutic interventions.

## AUTHOR CONTRIBUTIONS

Minwen Zheng designed the study. Yingjuan Chang and Ziliang Xu oversaw participant recruitment. Tian Zhang produced and organized the datasets. Chen Wang and Ziliang Xu conducted imaging data processing, and Peng Fang assisted with preprocessing and visual checking. Fan Guo performed the initial statistical analysis and wrote the first draft of the manuscript. Yuanqiang Zhu assisted in additional analyses and writing of the manuscript. Peng Fang and Minwen Zheng provided feedback on the study design during the study implementation. All authors have contributed to and approved the final manuscript.

## FUNDING INFORMATION

This study has received funding from the Key R&D Program Projects of Shaanxi, China (2022JM‐575), Boost Program of Xijing Hospital (JSYXM28, JSYXZ08, and XJZT21CM21), China Postdoctoral Science Foundation (2019M653963), and Military Medical Science and Technology Youth Training Program (20QNPY049).

## CONFLICT OF INTEREST STATEMENT

The authors have no conflicts of interest to declare.

## Supporting information


Figure S1.

Figure S2.

Figure S3.

Figure S4.

Figure S5.

Table S1.

## Data Availability

The data that support the findings of this study are not publicly available due to privacy restrictions but are available from the corresponding author upon reasonable request.
