# Supplementary figures and images for: White matter structural topologic efficiency predicts individual resistance to sleep deprivation
Source: CNS Neurosci Ther. 2023 Jul 5;30(2):e14349. doi: 10.1111/cns.14349 (PMC10848061; doi:10.1111/cns.14349)

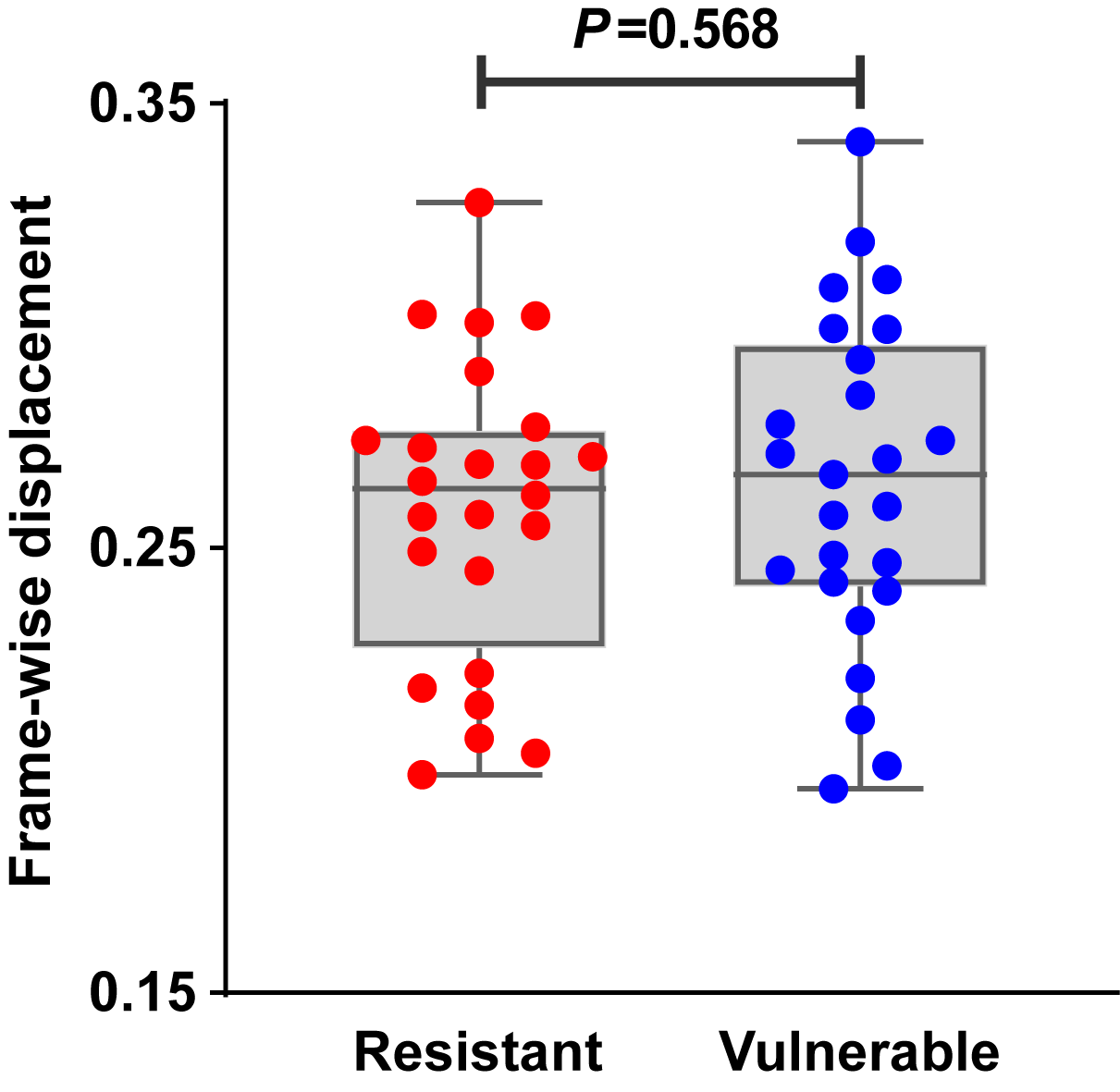

Supplement: Supplementary file 1 — Figure S1. [file CNS-30-e14349-s005.tif]

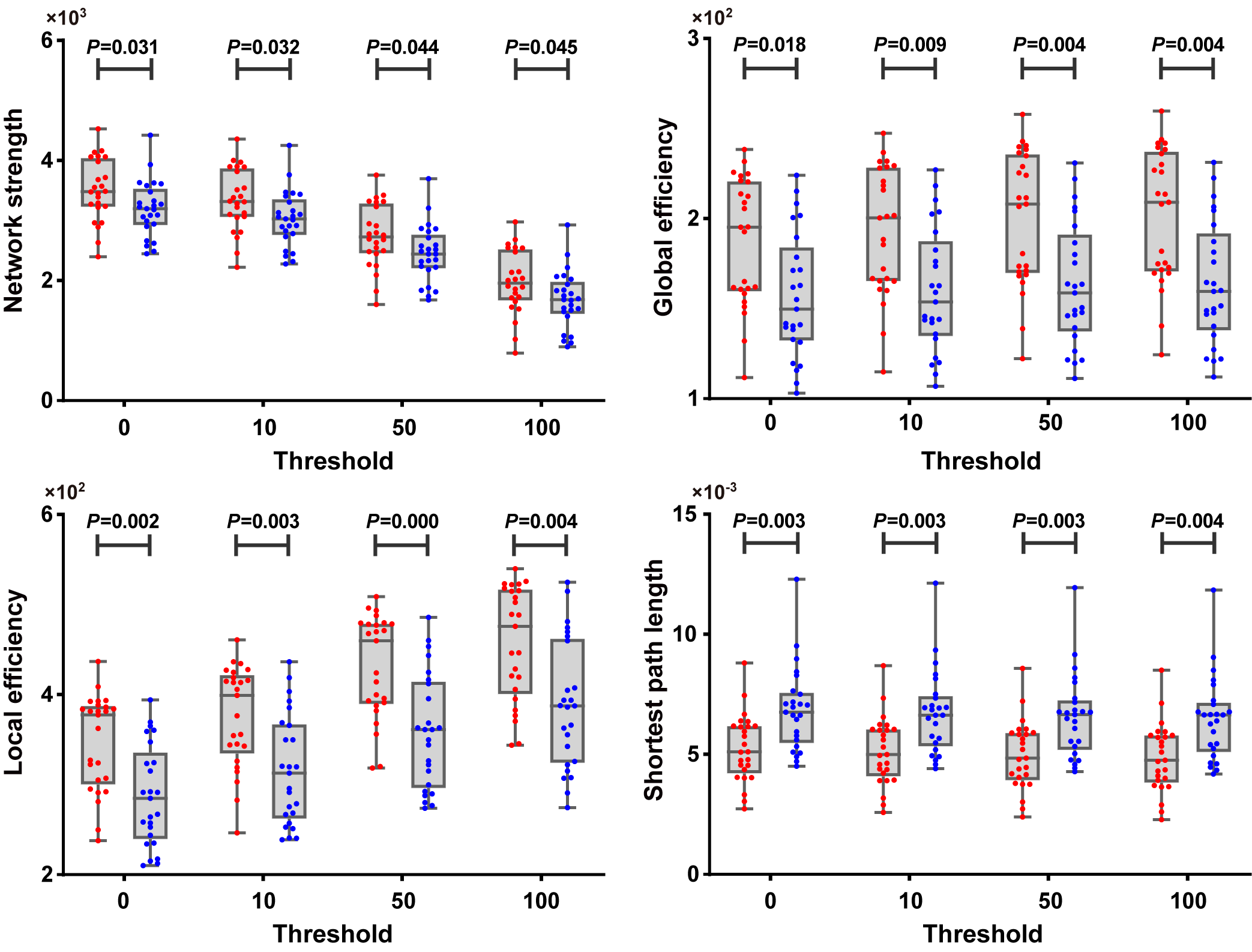

Supplement: Supplementary file 2 — Figure S2. [file CNS-30-e14349-s002.tif]

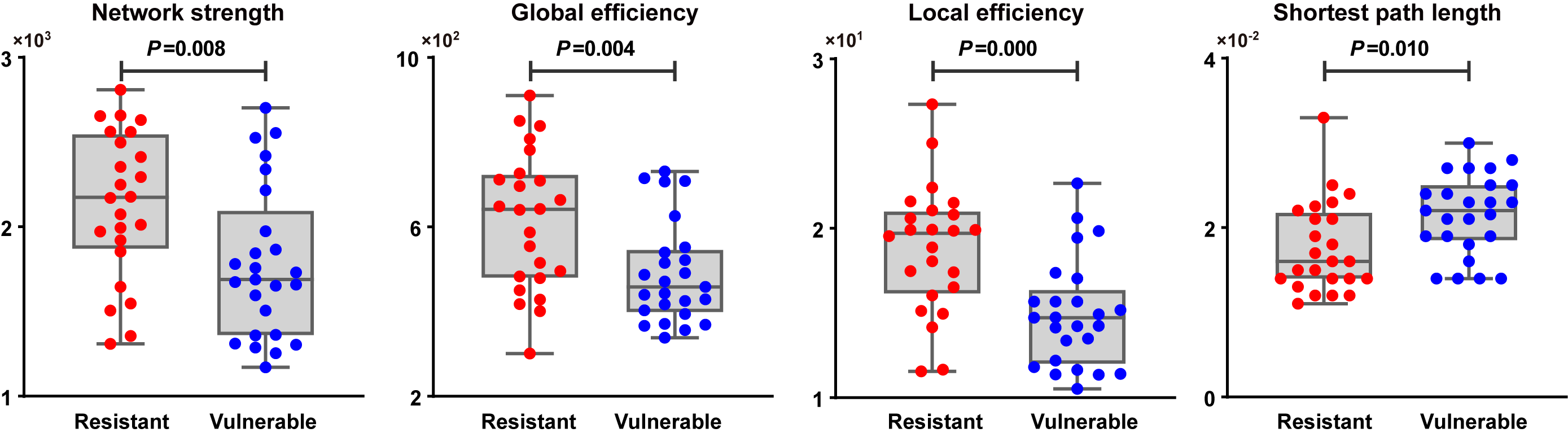

Supplement: Supplementary file 3 — Figure S3. [file CNS-30-e14349-s003.tif]

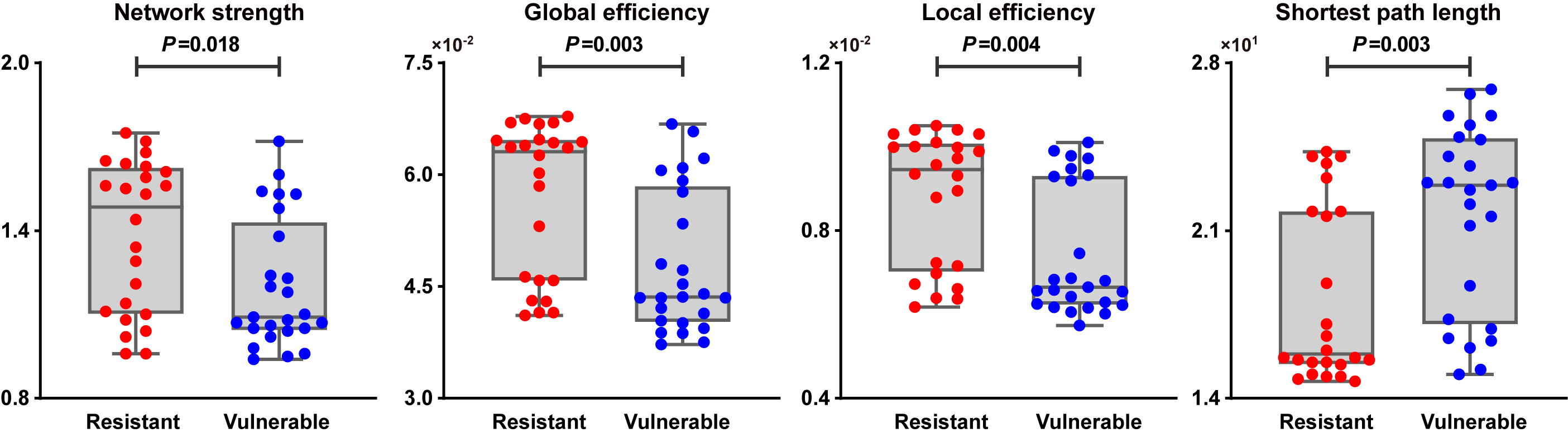

Supplement: Supplementary file 4 — Figure S4. [file CNS-30-e14349-s004.tif]

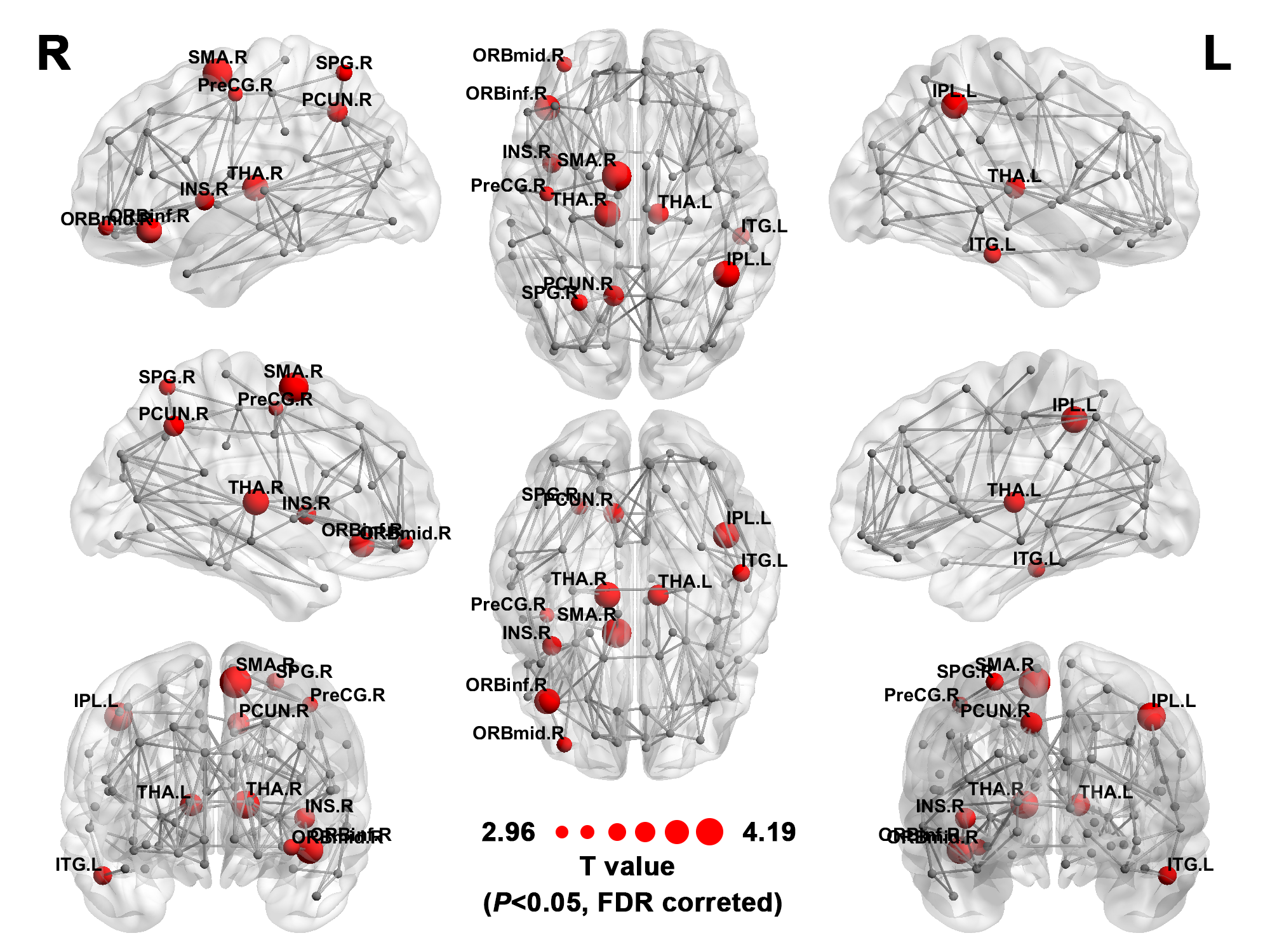

Supplement: Supplementary file 5 — Figure S5. [file CNS-30-e14349-s001.tif]
