# Supplementary material for: White matter structural topologic efficiency predicts individual resistance to sleep deprivation
Source: CNS Neurosci Ther. 2023 Jul 5;30(2):e14349. doi: 10.1111/cns.14349 (PMC10848061; doi:10.1111/cns.14349)
Supplement: Supplementary file 6 — Table S1. [file CNS-30-e14349-s006.docx]

| **Table S1.Nodal Efficiency differences between resistant and vulnerable to SD group (p < 0.05 FDR correction)** | | | | |
| --- | --- | --- | --- | --- |
| Regions | Resilience (n = 24) | Vulnerable (n = 25) | T Value | P Value |
| SMA.R | 0.055±0.011 | 0.043 ± 0.010 | 4.186 | <0.001 |
| IPL.L | 0.061±0.015 | 0.046 ± 0.011 | 4.058 | <0.001 |
| THA.R | 0.057 ± 0.013 | 0.045±0.009 | 3.839 | <0.001 |
| ORBinf.R | 0.065±0.014 | 0.052±0.010 | 3.823 | <0.001 |
| THA.L | 0.052 ± 0.012 | 0.042 ± 0.008 | 3.375 | 0.001 |
| PCUN.R | 0.070±0.017 | 0.056 ± 0.011 | 3.404 | 0.001 |
| INS.R | 0.068 ± 0.012 | 0.057 ± 0.012 | 3.295 | 0.002 |
| ITG.L | 0.049±0.012 | 0.040±0.008 | 2.96 | 0.005 |
| SPG.R | 0.063±0.015 | 0.052 ± 0.011 | 3.036 | 0.004 |
| ORBmid.R | 0.048±0.010 | 0.039±0.009 | 3.195 | 0.002 |
| PreCG.R | 0.065 ± 0.013 | 0.055 ± 0.012 | 3.077 | 0.003 |
| Data are presented as means ± standard deviation. The comparisons of nodal efficiency between groups were performed by using two-sample t tests.P<0.05 (FDR correction) indicated a significant group difference. SMA supplementary motor area, IPL inferior parietal, THA thalamus, ORBinf orbital part of inferior frontal gyrus, PCUN precuneus, INS insula, ITG inferior temporal gyrus, SPG superior parietal gyrus, ORBmid orbital part of middle frontal gyrus, PreCG precental gyrus. | | | | |
|  |  |  |  |  |
